# Household out-of-pocket payments for illness: Evidence from Vietnam

**DOI:** 10.1186/1471-2458-6-283

**Published:** 2006-11-15

**Authors:** Nguyen Thi Bich Thuan, Curt Lofgren, Nguyen Thi Kim Chuc, Urban Janlert, Lars Lindholm

**Affiliations:** 1 Planning and Financing Department of Ministry of Health, Hanoi, Vietnam; 2 Umeå International School of Public Health, Umeå University, Umeå, Sweden; 3 Public Health Faculty, Hanoi Medical University, Hanoi, Vietnam

## Abstract

**Background:**

In Vietnam, illnesses create high out-of-pocket health care expenditures for households. In this study, the burden of illness in the Bavi district, Vietnam is measured based upon individual household health expenditures for communicable and non-communicable illnesses. The focus of the paper is on the relative effect of different illnesses on the total economic burden of health care on households in general and on households that have catastrophic health care spending in particular.

**Methods:**

The study was performed by twelve monthly follow-up interviews of 621 randomly selected households. The households are part of the FilaBavi project sample – Health System Research Project. The heads of household were interviewed at monthly intervals from July 2001 to June 2002.

**Results:**

For the population in the Bavi district, communicable illnesses predominate among the episodes of illness and are the reason for most household health care expenditure. This is the case for almost all groups within the study and for the study population as a whole. However, communicable illnesses are more dominant in the poor population compared to the rich population, and are more dominant in households that have very large, or catastrophic, health care expenditure, compared to those without such expenditures.

**Conclusion:**

The main findings indicate that catastrophic health care spending for a household is not usually the result of one single disastrous event, but rather a series of events and is related more to "every-day illnesses" in a developing country context than to more spectacular events such as injuries or heart illnesses.

## Background

Ill health can have a significant economic impact on a household. Such an impact can trigger a spiral of asset depletion, indebtedness and reductions of essential consumption [1]. Health services may impose a regressive cost burden on households, especially in developing countries[2,3]. However, facing high user fees the poor may also restrict their utilisation of health services even to the extent that health expenses for the poor constitute a smaller share of income than for the rich[4]. The risk for very large (catastrophic) health expenditure has in some studies found to be larger for the rich The economic burden has two effects: first, the immediate loss of income due to absence from work; and second, large out-of-pocket expenses to cover the necessary medical care. Costly health care also deters people from using health services thereby generating prolonged or worsened health problems [5,6]. In addition, illness often place large intangible costs on households in terms of quality of life, discomfort and pain.

Evidence from Vietnam shows that the introduction of a new economic policy in 1986 increased the out-of-pocket health expenditures as a proportion of total health expenditures from 59% in 1989 [7], to 84% in 1998 [8,9]. The proportion of households facing catastrophic health expenditures and thereby risking impoverishment is also high (as much as 10% within one year) [10]. This is in contrast to official Vietnamese State policy that emphasises equity and free access to services for the poor [11,12].

In this paper, we focus the analysis on the relationship between types of illness and the burden of out-of-pocket health care payments on households in a rural district of Vietnam. The following questions are addressed:

• What is the household out-of-pocket health care expenditure associated with different types of illnesses?

• How does the health care expenditure differ among various socioeconomic groups?

• What are the illness types that are more likely to lead to catastrophic health care spending?

## Methods

The infrastructure of a demographic surveillance site in a district in northern Vietnam has been used for this study. Details of the district and the surveillance site are presented below. The sampling procedures and other methodological issues are also discussed.

### The setting and the interviews

This study was conducted in the Bavi district of Ha Tay province in Vietnam. Bavi district is situated in the northwest part of Vietnam, about 60 km west of Hanoi. It has a population of 235,000. There are three major ethnic groups in the district: Kinh (91%), Muong (8%) and Dao (1%). There are also some families of the Tay, Hoa and Khmer tribal groups. The district is divided into 32 communes including one small town. Farming is the predominant occupation. The average annual income in terms of rice production was about 290 kilograms/person/year in 1999, which corresponds to 50 USD per capita per year [13,14].

There is a demographic surveillance site in Bavi: The Epidemiological Field Laboratory for the Health Systems Research Project (FilaBavi) in Vietnam. FilaBavi is a joint project between Hanoi Medical University, Karolinska Institute, University of Umeå, and the Nordic School of Public Health in Sweden. The aims of the project are to implement a longitudinal epidemiological surveillance system to generate basic health and health care data, to supply information for health planning, serve as a background and a sampling frame for specific studies (especially intervention studies), and to constitute a setting for epidemiological training of research students. In 1999 a baseline household survey was conducted followed by quarterly surveillance of vital events and complete re-surveys every two years [14].

The infrastructure of FilaBavi was utilised for the study presented in this paper. The total FilaBavi sample consists of 11,089 households. The households were selected using a multistage sampling procedure. At the first stage, 67 population clusters were selected using probability proportional to size. These clusters had 11,089 households and 51,024 individuals [14]. Assuming α level of 5% and 50% probability that a household will have an episode of illness in a year, the required sample size becomes 576. To ensure adequate sample size, one out of every 18 households was randomly selected from the original sample for the purpose of this study. The procedure generated a sample of 629 households.

The study units of the FilaBavi project are households. The heads of households were interviewed at monthly intervals during July 2001 to June 2002. If the head of the household could not be contacted, another adult was interviewed. These household representatives provided information on the household's health situation, health care utilisation, health expenditures and total expenditures. For information on illnesses the respondents were specifically asked if the household member in question had seen a medically trained person (doctor, nurse, health worker or such) and if so, which diagnose had been made. For all questions related to female-specific diagnoses, the interviewers were instructed to interview the patient directly.

Households kept daily notes of their health situation and health care payments including illness events of every person in the household. During the first week of each month, the interviewer conducted an interview based on the daily notes from the previous month. The interviews were carried out by 42 qualified interviewers employed by the larger FilaBavi project. All interviewers had completed high school education and were inhabitants of the Bavi district. The interviewers used a structured questionnaire and were given special training on data collection strategies for collecting information on income, expenditure and illnesses. Ten per cent of the questionnaires were randomly selected for re-interviews before the data entry.

### Illnesses

This study defines an illness episode as a report of at least one of the following conditions: having stayed in bed; having been restricted from normal activities (e.g. work, school); having been able to do normal activities but with reduced capacity for at least one day and/or having to pay out-of-pocket for health services. An illness episode is concluded when normal activities (with normal capacity) are resumed. All episodes of illness that occurred during the month prior to the interview were recorded. An individual could thus have several illness episodes in a month [15].

In the interview questionnaire, respondents were specifically asked whether members of the household had the following symptoms or illnesses: cough; fever; difficulty breathing; headache; abdominal pain; intestinal disorder; pain in bones or joints; injury/accident; hypertension; heart illness. Respondents were also asked to specify whether members of the household had any other symptoms or illnesses.

From the respondents' answers, illnesses were classified into communicable, non-communicable, other and mixed illnesses.

Communicable illnesses include respiratory infection (throat, flu, and cough), fever, diarrhoea and the following symptoms/illnesses when combined with fever: abdominal pain; pimples; illnesses of the teeth, liver, kidney, eyes or skin; difficulty breathing; headache; stomach ache; gynaecological problems.

*Non-communicable illnesses *include diabetes, goitre, cancer, neurological problems, rheumatologic problems, hypertension, heart illness, allergic problems, and the following symptoms/illnesses when not combined with fever: illnesses of the liver, kidney, eyes, or skin; difficulty breathing; headache; stomach ache; gynaecological problems.

*Other illnesses *include symptoms/illnesses not included in the above list, such as toothache without fever, vertigo etc.

*Mixed illnesses *include symptoms/illnesses belonging to two or more of the above categories.

### Expenditures and the classification of income groups

The expenditures recorded in the study are the total financial outlays that the households had each month for food, health care and other means. Health care payments include medical expenses (for consultations, tests, x-rays, drugs and beds) as well as non-medical expenses (for travel, food and other related means).

The households were classified according to their economic situation in two ways. First, total household expenditure quintiles were used. Expenditures, rather than income, are commonly used as a measure of socio-economic status in developing countries for several reasons. First, household expenditures tend to vary less than income, and second, households may be less willing to state their true income or may underestimate their total income [14].

In order to test the robustness of our expenditure classifications we also used a socioeconomic classification determined by local leaders. Households were ultimately classified into rich, medium and poor based on standards set by the Ministry of Labour, Invalids and Social Affairs.

### Catastrophic health care spending

To identify the households that had catastrophic health care spending we used the methodology developed by Xu et al [16-18]. According to this approach, catastrophic spending occurs when health care expenditure for a household exceeds 40% of the households' capacity to pay.

A household's capacity to pay (*CTPi*) was calculated in the following way:

*CTP*_*i *_= *TEXP*_*i *_- *SE*_(45–55)*i *_If *FEXP*_*i *_≻ *SE*_(45–55)*i*_

*CTP*_*i *_= *TEXP*_*i *_- *FEXP*_*i *_If *FEXP*_*i *_≺ *SE*_(45–55)*i*_

*TEXP *denotes total expenditure and *FEXP *denotes food expenditure. *SE *stands for subsistence expenditure and is the average food expenditure for households whose food expenditure share of total expenditure is in the 45^th ^to 55^th ^percentile.

Microsoft ACCESS was used for data entry and data analyses were performed using SPSS software.

## Ethical Considerations

This specific study was approved by the Scientific and Ethical Committee for Medical Research, Hanoi Medical University, and also received approval from the Ministry of Health (Decision No: 1089-QD-BYT-2001) and local authorities, as well as from heads of households.

## Results

The monthly repeated interviews were completed for 621 of the 629 households selected for the study. The survey could not be completed for 6 households that migrated from the Bavi district and for 2 households that declined to take part in the study. There were a total of 2,727 individuals in the 621 households surveyed (table 1). Average household size was 4.4 persons. The household size was significantly lower in the bottom expenditure quintile compared to the other quintiles.

More than 90% of individuals suffered from at least one episode of illness during the year (table 1) and there was no difference in illness incidences among the expenditure quintiles". However, the number of illness episodes per person is higher in the bottom expenditure quintile compared to the other quintiles. Drugs and/or services were used in 97% of the illness episodes. However, this includes self-treatment (a person buying drugs without prescription and without any professional advice). In the lower expenditure quintiles, the share of episodes where no drugs or services were used is larger than in the higher quintiles.

The episodes are classified according to the respondents' self-reported illnesses. Communicable illnesses predominate in the study and were reported for almost two thirds of the episodes. Non-communicable illnesses account for approximately one fourth of the episodes and the remaining tenth of the episodes is comprised of injuries, other illnesses and a mix of illnesses. Communicable illnesses are a little less, and non-communicable illnesses a little more common in the top expenditure quintile compared to the other quintiles for these illness categories (table 2). This difference between socioeconomic groups also appears when using the local leaders' classification of households – the poor report more communicable, and less, non-communicable illnesses.

The expenditures for different illnesses show the same pattern as for episodes. Expenditure for communicable illnesses account for half of the total curative expenditures in the top total household expenditure quintile compared to two thirds in the other quintiles. A similar difference is found when comparing rich and poor according to local leaders' classification. The poor pay a larger share of their curative health expenditure for communicable illnesses than the rich do. However, expenditure on communicable illnesses still dominates in all groups (See table 3)

In table 4, health care expenditures are related to household capacity to pay for health care (see the method section above for a description of this concept). There are seven households that had catastrophic health care expenditure, defined as health care expenditure being more than 40% of the households' capacity to pay. For these households, expenditures for communicable illnesses account for as much as 85% of total health care expenditure. For the large majority of households (502 of the 621 households) health care expenditure is equal to or smaller than 10% of their capacity to pay. In this group communicable illnesses still dominate but constitute a smaller share of health expenditure than in the other groups, with one notable exception. In the group of twelve households having health expenditure between 30% and 40% of households' capacity to pay, expenditure for non-communicable illnesses corresponds to 59% of their total health care expenditure. However, further examination of those twelve households (data not shown here) shows that spending for communicable illnesses predominates for seven households, non-communicable illnesses predominate for three, and the remaining two have communicable and non-communicable illness expenditures of approximately the same size. The reason why expenditures for non-communicable illnesses predominate for the total group is that there was one household with such a large expenditure for non-communicable illnesses that it outweighs the predominance for communicable illnesses in the majority of households in the group.

There are seven households whose health expenditures exceeded 40% of their capacity to pay. Their shares of expenditures for the different illnesses are given in table 5. Communicable illnesses predominate for five of the households, non-communicable illnesses for one of the households, and injuries for the remaining household. Several interesting observations are seen for the seven households with catastrophic health care spending (table 5). The number of household members is relatively small. All, but one of them, have 1 or 2 members. The average for all households is 4.4 members (compare to table 1). By contrast, the number of illness episodes per person having been ill is relatively large. The average for this group of seven households is 6.1 episodes compared to 3.3 episodes for all households in the study.

The most costly episode is a heavy burden for all of the households. However, for only two of the households does it constitute more than half of health expenditures. For the other households the most costly episode makes up between 23% and 46% of health expenditures. When measured by local leaders' classification of households into rich and poor, few of the households with catastrophic health care spending are poor. However, if household wealth is judged by total household expenditure quintiles, five of the seven households are found in the bottom quintile.

In summary, the main finding in this study is that communicable illnesses predominate among the episodes of illness as well as for household health care expenditure in Bavi district. This is the case for almost all studied groups in this study and for the studied population as a whole. However, communicable illnesses are more dominant in the poor population compared to the rich population, and more predominant for those households that have very large, or catastrophic, health care expenditure, than for other households.

The main finding in this study thus indicates that catastrophic health care spending for a household is not usually the result of one single disastrous event, but rather a series of events, and is related more to "every-day illnesses" in a developing country context than to more spectacular events such as injuries and heart illnesses.

## Discussions

A particular strength of this study is that the survey was performed by trained and experienced interviewers living in the Bavi district with a good knowledge of the local population. Nevertheless, there are several possible weaknesses of this study, which are discussed below.

Our data are likely to underestimate the burden of illness for several reasons. We have only measured the household out-of-pocket expenditures for health care. The income losses, or time costs, due to illness that are incurred by households have not been measured. These losses are presumably large in comparison to out-of-pocket expenditures, particularly for households that have faced serious illnesses. In a study from Burkino Faso it was found that time costs were as high as 73% of total household costs due to illness [19]. However, we have limited the scope of this study to out-of-pocket health care payments since these are considered to be a relatively large problem in Vietnam compared to many other countries [18,20]

A further reason why our data on health expenditure are likely to underestimate the burden of illness is that high out-of-pocket expenditure restricts access to health care. Drugs or services have been used for almost all of the episodes of illness recorded in this study (table 1). However, this includes self-treatment, which is likely to be a low cost alternative to the preferred medical treatments that would be selected if there were no financial restrictions. There is also uncertainty associated with the ways we measure who is rich and poor among the studied households. We have used total household expenditure quintiles to discriminate between different groups. This is common in developing country studies. When interviewing households, expenditures ("effective income") are believed to give a more accurate picture of the household economy than income, reasons for which have already been stated in the method section above. However, studies have also shown that the correlation between different measures of households' economy may not be high [3]. Therefore, we have compared the total expenditure quintiles to a classification of socioeconomic status carried out by local leaders in Bavi. In most instances the conclusions are not altered when using one classification or the other. In one case, however, the classification method makes an important difference. Five of the seven households in the study that have catastrophic health care spending, belong to the bottom total expenditure quintile and only one belongs to the top quintile. However, according to the local leaders' classification, two of these households are rich, three are classified as medium and only two are poor. In this case we have refrained from drawing any conclusions concerning the socioeconomic status of the households with catastrophic health expenditures. But in other cases, where the two methods of classification point in the same direction we believe our conclusions are valid.

The illnesses in this study have been classified into five broad groups. Our data do not allow a more detailed classification. We find that household out-of-pocket expenditure for health is dominated by communicable illnesses in the Bavi district of Vietnam. Similar findings have been reported when particular illnesses have been at focus. In a recent review of the household economic burden of illness in developing countries[21] it was found that costs for tuberculosis and HIV/AIDS in many cases were catastrophic. The latter concept was in that study defined as direct costs being more than 10% of income. In a Nigerian study of malaria holo-endemic areas it was found that costs for this disease were almost 50% of total household curative costs [22]. Another study has addressed the so called neglected diseases, i.e. parasitic diseases for which there is a relatively low interest both from the health services sector and research[23]. However, the household costs for such diseases are reported to be very high. In a study from Burkino Faso expenditures for all illnesses are covered and subdivided according to the Global burden of disease classification [24]. The illnesses/diseases are ranked by the total out-of-pocket expenditures they have caused for households. Of the ten most costly diseases six are communicable diseases, three are non-communicable diseases and one consists of injuries.

In this light our findings on the very large, or catastrophic, health care expenditures are interesting. The word "catastrophic" easily leads the mind to single disastrous events, such as an accident or a stroke, rather than to a chain of more "everyday" illnesses. However, our data indicates this may not be so. Communicable illnesses dominate strongly for the households that have catastrophic spending. The number of episodes of illness is relatively large and the most costly episode is, for most households, less than half of total health care expenditure (in fact it is considerably lower in most cases). This paints a picture of households not being catastrophically thrown into poverty by a sudden disaster but rather grinded into poverty by a longer sequence of events.

## Competing interests

We, the authors, declare that there are no financial or non-financial competing interests (political, personal, religious, academic, ideological, intellectual, commercial or any other).

## Authors' contributions

We are co-authors in this paper. Each author has participated sufficiently in the work to take public responsibility for appropriate portions of the content as follow: TNTB: The first author, who designed the questionnaire, was responsible for and monitored the interview process, performed the statistical analysis, and drafted the manuscript CL: The second author, who participated in the planning of the study, took part in the statistical analysis and was involved in drafting the manuscript and revising it. CNTK and LL: The third and the fifth authors, who have participated in the planning of the study, and in the revisions of the manuscript. UJ: The fourth author, who has participated in the statistical analysis and particularly in the classification of illnesses. All of us, the co-authors, read and approved the final manuscript.

## Pre-publication history

The pre-publication history for this paper can be accessed here:



## Figures and Tables

**Table 1:** Persons suffering from illness and the number of illness episodes during the year.

	Household total expenditure quintiles					Total
	1 (bottom)	2	3	4	5 (top)	
Number of persons	377	576	567	600	607	2727
Number of household members	3.0	4.7	4.6	4.8	4.9	4.4
Percent of persons suffering from at least one episode of illness	93.6%	92.2%	93.3%	92.5%	93.3%	92.9%
Total number of illness episodes	1 303	1 749	1 668	1 929	1 731	8 380
Episodes of illness per person having suffered at least one episode of illness	3.7	3.3	3.2	3.5	3.1	3.3
Percent of illness episodes where no drugs or services were used	4.5%	3.5%	3.0%	2.7%	1.9%	3.0%

**Table 2:** The percent of illness episodes for each illness group.

	Local leaders' classification			Household expenditure quintiles					Total
			
	Poor	Medium	Rich	1 (bottom)	2	3	4	5 (top)	
	%	%	%	%	%	%	%	%	%
Injuries	3.3	3.3	3.3	4.6	2.7	2.9	3.1	3.4	3.3
Communicable illnesses	65.1	60.9	59.3	62.4	62.7	60.9	62.7	58.4	61.4
Non-communicable illnesses	22.6	26.5	28.6	22.8	25.4	26.8	24.9	30.3	26.2
Other illnesses	1.5	1.4	1.2	1.2	1.4	1.0	1.7	1.6	1.4
Mix of illnesses	7.5	8.0	7.7	9.1	7.8	8.5	7.5	6.5	7.8
Total	100.0	100.0	100.0	100.0	100.0	100.0	100.0	100.0	100.0
Number of episodes	1 712	4 707	1 961	1 303	1 749	1 668	1 929	1 731	8 380

**Table 3:** Household curative health expenditures for different illness groups

	Local leaders' classification			Household expenditure quintiles					Total
			
	Poor	Medium	Rich	1 (bottom)	2	3	4	5 (top)	
	%	%	%	%	%	%	%	%	%
IInjuries	3.0	2.6	2.1	5.4	2.0	2.3	1.5	2.7	2.5
Communicable illnesses	68.3	58.4	60.7	61.5	66.2	64.9	64.5	51.5	60.6
Non-communicable illnesses	22.8	28.6	28.7	24.2	22.8	23.5	25.9	35.7	27.7
Other illnesses	1.0	1.3	1.3	1.2	1.0	0.9	1.2	1.7	1.2
Mix of illnesses	5.0	9.2	7.2	7.7	8.1	8.5	6.9	8.5	8.0
Total	100.0	100.0	100.0	100.0	100.0	100.0	100.0	100.0	100.0
Curative health expenditure, VND*	356 218	462 945	607 179	243 149	401 942	440 098	565 825	713 987	472 947

Total health expenditure**, VND*	378 506	496 978	703 257	258 994	430 735	487 042	607 638	808 298	518 491
Total household expenditure, 1000 VND*	7 969	11 628	19 886	3 559	6 932	9 637	14 064	29 421	12 718
Tot household exp/Tot household exp	4.7%	4.3%	3.5%	7.3%	6.2%	5.1%	4.3%	2.7%	4.1%

*VND = Vietnamese dong

** The difference between curative health expenditure and total health expenditure are household expenses for rehabilitation, family planning services and health insurance.

**Table 4:** The percent of episodes and health expenditure for different illnesses related to households' capacity to pay

	The ratio (*x*) of total household health expenditures to household capacity to pay											
	*x *≤ 10%		10% <*x *≤ 20%		20% <*x *≤ 30%		30% <*x *≤ 40%		40% <*x*		All	
						
	Episodes %	Expenditure %	Episodes %	Expenditure %	Episodes %	Expenditure %	Episodes %	Expenditure %	Episodes %	Expenditure %	Episodes %	Expenditure %
						
Injuries	3.3	3.4	3.3	2.2	3.5	0.5	4.0	0.7	1.3	2.1	3.3	2.5
CD	59.8	58.6	67.6	62.9	63.4	72.9	62.4	36.2	69.6	85.0	61.4	60.6
NCD	27.8	29.0	20.1	19.5	23.9	23.4	23.5	58.6	20.3	11.1	26.2	27.7
Other	1.4	1.8	1.3	1.0	1.6	0.3	1.3	0.3	-	-	1.4	1.2
Mix	7.8	7.3	7.8	14.4	7.6	2.9	8.7	4.2	8.9	1.8	7.8	8.0
Total	100	100.0	100	100.0	100	100.0	100	100.0	100	100.0	100	100.0
No of episodes	6 423		1 415		314		149		79		8 380	
Average house-hold health exp		304 612		855 601		1 617 974		2 072 108		2 267 829		472 947
No. of house-holds	502		81		19		12		7		621	

CD = communicable illnesses, NCD = non-communicable illnesses, Other = other illnesses, Mix = mix of illnesses

**Table 5:** The share of expenditure for different illnesses for the seven households whose health care expenditures exceed 40% of their capacity to pay

Illnesses	HH 1	HH 2	HH 3	HH 4	HH 5	HH 6	HH 7	All
Health care expenditure as a percentage of capacity to pay	41%	46%	49%	49%	55%	60%	83%	

Injuries	0.0%	0.0%	46.0%	0.0%	0.0%	0.0%	0.0%	2.1%
Communicable illnesses	39.2%	93.4%	20.4%	59.9%	87.7%	87.4%	94.7%	85.0%
Non-communicable illnesses	59.1%	2.6%	33.3%	10.2%	12.3%	12.2%	0.0%	11.1%
Other illnesses	0.0%	0.0%	0.0%	0.0%	0.0%	0.0%	0.0%	0.0%
Mix of illnesses	1.7%	4.0%	0.3%	29.9%	0.0%	0.4%	5.3%	1.8%
Total %	100.0%	100.0%	100.0%	100.0%	100.0%	100.0%	100.0%	100.0%
Total curative expenditure, 1000 VND	406	3 785	727	167	7 834	1 646	1 310	15 875
Total expenditure 1000 VND	1 344	9 668	1 842	923	24 847	3 336	2 501	44 460
Total health expenditure, 1000 VND	406	3 785	727	177	12 507	1 646	1 461	20 709
Health exp/tot exp	30.2%	39.2%	39.5%	19.2%	50.3%	49.3%	58.4%	46.6%
No of episodes	11	10	13	8	15	10	12	79
No of HH members	1	2	2	1	4	2	1	13
No of persons in HH being ill	1	2	2	1	4	2	1	13
Episodes per person being ill	11	5	6.5	8	3.8	5	12	6.1
Income group*	Medium	Rich	Poor	Medium	Rich	Poor	Medium	
Total expenditure quintile	1	3	1	1	5	1	1	
Most costly episode, 1000 VND	124	3 403	334	50	1 779	1 230	470	
Most costly episode as a percentage of curative expenditure	30.5%	89.9%	46.0%	29.9%	22.7%	74.7%	35.9%	

* According to local leaders' classification

VND = Vietnamese dong
